# Comparison of Six-Week, Three-Month, and One-Year Postoperative Clinical Results of Kinematic and Mechanical Alignment in Primary Medial Pivot Total Knee Arthroplasty

**DOI:** 10.7759/cureus.64517

**Published:** 2024-07-14

**Authors:** Fabian Reinisch, Aikaterini Ioannou, Alex Eberle, Markos Ioannou

**Affiliations:** 1 Pathology, Stadtspital Triemli, Zürich, CHE; 2 Medicine and Surgery, University of Patras, Patras, GRC; 3 Surgery, Zollikerberg Hospital, Zollikerberg, CHE; 4 Orthopaedic Surgery, Zollikerberg Hospital, Zollikerberg, CHE

**Keywords:** knee replaecment, osteoarthritis (oa), kinematic alignment, mechanical alignment, total knee replacement (tkr)

## Abstract

Background: Total knee replacement (TKR) is a common surgical solution for severe osteoarthritis. Kinematic alignment (KA) and mechanical alignment (MA) are two popular techniques. There is ongoing debate over the optimal method, influenced by varying long-term results and a scarcity of data on short-term postoperative outcomes. Early evaluation of these techniques is vital for improving rehabilitation outcomes and ensuring patient satisfaction.

Methods: This study retrospectively analyzed outcomes from 71 KA-TKRs and 85 MA-TKRs performed between 2019 and 2021. Knee flexion, visual analog scale (VAS) scores, EuroQol-5d (EQ-5d) quality of life measures, and dependence on walking aids were evaluated. Evaluations were conducted at baseline, six-weeks, three-months, and 12-months postoperatively using two-sample t-tests for continuous data and Pearson’s chi-squared test for categorical data.

Results: At six-weeks and three-months postoperatively, the KA group exhibited significantly better outcomes in knee flexion (98.6° vs. 90.2° at six-weeks; 114.7° vs. 94.2° at three-months), pain management, and reduced walking aids compared to the MA group. By 12-months, these differences were no longer significant, with both groups showing comparable results in knee flexion, pain scores, and patient-reported outcomes.

Conclusion: KA offers substantial short-term advantages over MA for pain relief, increased knee flexion, and independence from walking aids. However, these benefits do not persist at one-year post-surgery, indicating a convergence of outcomes between the two techniques. Larger studies with extended follow-ups are required to determine the long-term implications of these alignment strategies.

## Introduction

Osteoarthritis is the most widespread joint disease, significantly contributing to disability in the elderly, with the knee being one of the most affected joints [1]. Total knee replacement (TKR) is recognized as an efficient and cost-effective treatment aiming to relieve pain, improve functional mobility, and enhance overall patients’ quality of life [2].

Traditionally, mechanical alignment (MA) has been considered the gold standard for TKR. In MA, the prosthesis is aligned to achieve a neutral hip-knee-ankle axis, ignoring the patient’s pre-arthritic varus or valgus conditions. However, dissatisfaction rates among patients undergoing MA TKR can be as high as 20%. The prevalence of residual symptoms and post-operative complaints also show a wide range of incidence rates, ranging from 33-85%, according to various studies in the literature [3-6].

With advancements in technology and software, newer alignment techniques have been developed. They include preoperative examinations such as 3D imaging with integrated computer software to determine the hip-knee-joint-leg axis [7]. Kinematic alignment (KA) aims to restore the patient-specific anatomy of the pre-arthritic state through co-alignment of the three kinematic axes and joint lines of the native joint, with minimal to no ligament release required. This method offers the benefits of a more balanced joint capsule due to natural ligament tension, preserved soft tissue integrity, and reduced pain [7-9,10].

Currently, there is no consensus on the optimal alignment technique for TKR. Existing studies frequently yield conflicting results, primarily focusing on outcomes observed one to two years post-surgery [11,12]. Furthermore, there is a scarcity of research detailing direct comparisons between KA and MA in the early rehabilitation phase within the first year post-operation [13].

In this retrospective study, our objective is to compare the outcomes of KA-TKR with those of MA-TKR at six-week, three-month, and 12-month follow-ups. We hypothesize that KA TKR will result in significant reductions in pain and enhanced functional improvements, including range of motion, reduced use of walking aids, and better scores on patient-specific measurement tools during the early rehabilitation period compared to MA TKR.

Our findings are expected to offer valuable insights for clinicians in optimizing postoperative recovery strategies, thereby enhancing patient care, and informing future clinical guidelines.

## Materials and methods

This study is a single-center, retrospective analysis comparing two anonymous cohorts who underwent total knee replacement (TKR) in 2019 and 2021. In 2019, the mechanical alignment (MA) technique was used for 85 patients who received medial pivot knee arthroplasty for primary osteoarthritis. In 2021, the surgical protocol was updated to the kinematic alignment (KA) technique, with 71 patients undergoing the same procedure. All participants provided informed consent, and none of the patients were lost to follow-up. A standardized surgical approach was employed, using a mid-vastus split technique and consistent prosthetic components. One experienced surgeon performed all the surgeries. To further reduce potential bias, patients from the transition year 2020 were not included in the study. This temporal distinction between the use of MA in 2019 and KA in 2021 allows for a direct comparison of the outcomes between the two alignment techniques, effectively contrasting the advantages of each based on evolving knowledge and promising research findings.

Outcomes were assessed at six-weeks, three-months, and 12-months postoperatively. The six-week and three-month outcomes were assessed using the Visual Analogue Scale (VAS) pain score (ranging from 0 to 10, with higher scores indicating worse pain), active flexion (measured in degrees), and the use of walking aids (yes/no). Active flexion was measured with a long-arm goniometer with the patient in a supine position. The use of walking aids was determined based on their usage during outdoor activities, even if they were only used occasionally for safety purposes. Furthermore, at the 12-month follow-up, we administered the modified EQ-5d survey (EQ-5d index: 0-1: 0=worst health/1=full health), a patient-related measurement tool for evaluating health-related quality of life [14].

A cemented medial pivot prosthesis was used in all patients (GMK Sphere, Medacta, Switzerland). The concept of computer tomography (CT)-based patient-specific instrumentation begins with a standardized CT protocol of the hip, knee, and ankle. Using the software, a 3D model of the knee was created, and either MA or KA was planned. The guides, which create the bone cuts, are designed to fit into the arthritic knee. In both, MA and KA, the femoral and tibial cutting blocks are positioned extramedullary. The patient-specific guides were sterilized according to the manufacturer’s instructions, and opened within the sterile field, while the specific patient identifiers on the guides were visually confirmed. The cartilage had to be removed from the fitting position of the guides, as demonstrated by the 3D model. This is mostly achieved with electrocoagulation or a scalpel. The guides are made to fit the knee in one specific position according to its arthritic anatomy. Both cruciate ligaments were removed according to the operative technique of medial pivot TKR. The distal femoral cut was made through the slot of the patient-specific guide. The conventional 4-in-1 cutting block (GMK efficiency) according to the pre-planned size of the femoral component, was placed into the two-guide pinholes in the distal femoral articular surface, to perform the femoral cuts. Osteophytes of the posterior femur were removed manually with a curved chisel, and the posterior capsule was released. The tibial patient-specific guide was then secured by drilling two pins through the pinholes on the proximal surface of the tibial guide, and the tibial cut was made through the slot in the guide. Subsequently, the trial components were placed, and the range of motion, stability, rotation of the components, patellar tracking, and flexion-extension gaps were checked. 

In mechanically aligned TKR the planning was performed to achieve a neutral coronal mechanical limb alignment with the angle of the distal resection set at 5° valgus and the posterior femoral cuts with a 3° external rotation. According to the surgeon’s assessment, to provide optimal patella tracking and to balance the flexion and extension gaps, a collateral and retinaculum ligament release was performed, where necessary.

In patient-specific KA planning, the algorithm reconstructs a 3D model of the normal, pre-arthritic, knee. The software fills articular defects and equalizes the gap between the medial and lateral compartments, to restore the joint line to the pre-arthritic state. The axes and joint lines of the components were co-aligned with the three kinematic axes and joint lines of the pre-arthritic knee, without any ligament release. However, the medial and lateral capsules were released at the margins of the menisci and the posterior capsule was released in all cases to remove osteophytes of the posterior capsule. The definitive components were placed with cement. Drainage was set in all cases and removed on the first postoperative day. Post-operative management was identical for both groups, with a standardized physiotherapeutic protocol. Full weight bearing was allowed from the first postoperative day in both groups. 

Statistical analysis was conducted using the R statistical software program. Two-sample t-tests were employed for continuous variables following a normal distribution to assess any significant differences. Categorical variables, such as the usage of walking aids, were evaluated using Pearson's chi-squared test. 

Differences in pain scores, mean flexion, and walking aid usage at six-weeks and three-months post-operation were analyzed. Additionally, 12-months postoperative, further comparison was conducted, focusing on the changes in mean flexion, pain scores, and the EQ-5d health index values. A p-value of < 0.05 was considered statistically significant.

## Results

The preoperative assessment revealed no significant differences in demographic and clinical parameters, including age, gender, body mass index (BMI), and EQ-5d index, between the 2019 and 2021 cohorts (Table 1). In contrast, the six-week postoperative interval indicated multiple significant statistical differences. Compared to the 2019 cohort, the 2021 group showed a higher mean flexion (98.6° vs. 90.2°, p<0.001; Table 2), a significant reduction in pain scores (Kinematic Alignment 2.78 vs. Mechanical Alignment 3.81, p<0.001) and a lower need for walking aids (KA 63.4% vs. MA 81.2%, p=0.018).

**Table 1:** Baseline and clinical characteristics comparing kinematic and mechanical alignment Abbreviations: ASA = American Society of Anesthesiologists (ASA) score, BMI = body-mass-index, EQ-5d-index = EuroQol-5D-Index, N= number, SD = standard deviation, p < 0.05 = statistically significant.

Variables	Kinematic Alignment	Mechanical Alignment	p-value
N	71	85	Not evaluated
Women (%)	58	61	Not evaluated
Mean age±SD	71±10	72±9	0.516
Mean BMI±SD	27.2±5.9	27.5±6.8	0.768
Morbidity State N (%)			
ASA1	5 (7%)	5 (6.2%)	Not evaluated
ASA2	36 (50.7%)	36 (44.8%)	Not evaluated
ASA3	30 (42.3%)	37 (46.2%)	Not evaluated
ASA4	0 (0%)	3 (2.8%)	Not evaluated
ASA5	0 (0%)	0 (0%)	Not evaluated
Mean EQ-5d index 0-1 (0=worst health / 1=full health)±SD	0.63±0.2	0.64±0.2	0.771
Mean Pain score 0-10 (0=no pain / 10=worst pain)±SD	7.40±1.8	7.35±1.8	0.863
Mean flexion in degrees (°)±SD	112±12.3	115±12.8	0.139

**Table 2:** Comparison between the kinematic and mechanical alignment at the six-week follow-up Abbreviations: SD = standard deviation. p < 0.05 = statistically significant.

Variables	Kinematic Alignment	Mechanical Alignment	t-value	p-value
Pain score 0-10 (0=no pain / 10=worst pain)±SD	2.78±1.6	3.81±1.7	-4.17	<0.001
Mean flexion in degrees (°)±SD	98.6±11.2	90.2±8.1	5.83	<0.001
			Chi-square	p-value
Use of walking aids Yes: N (%)	45 (63.4%)	69 (81.2%)	6.10	0.013

Three months after surgery, the KA cohort presented significantly superior outcomes compared to those of the MA group, including lower pain scores (KA 1.12 vs. MA 2.95, p<0.001), greater mean flexion (KA 114.7° vs. MA 94.2°, p<0.001; Table 3), and decreased dependence on walking aids (KA 11.3% vs. MA 32.9%, p=0.002).

**Table 3:** Comparison between the kinematic and mechanical alignment at the three-month follow-up Abbreviations: SD = standard deviation. p < 0.05 = statistically significant

Variables	Kinematic Alignment	Mechanical Alignment	t-value	p-value
Pain score 0-10 (0=no pain / 10=worst pain)±SD	1.12±1.5	2.95±1.8	-7.41	<0.001
Mean flexion in degrees (°)±SD	114.7±9.9	94.2±12.3	11.13	<0.001
			Chi-square	p-value
Use of walking aids Yes: N (%)	8 (11.3%)	28 (32.9%)	9.92	0.002

At the 12-month evaluation, both the KA and MA groups showed improvements in pain scores, EQ-5d index scores, and mean flexion compared to previous follow-ups. Pain scores were comparable between the KA and MA groups (KA 1.0 vs. MA 0.98, p=0.934; Table 4), as well as the mean flexion (KA 123° vs MA 122°). Similarly, there were no significant differences between the groups in the EQ-5d index (KA 0.90 vs. MA 0.91, p=0.535).

**Table 4:** Comparison between the kinematic and mechanical alignment at the twelve-month follow-up Abbreviations: EQ-5d-index = EuroQol-5d-Index, SD = standard deviation. p < 0.05 = statistically significant.

Variables	Kinematic Alignment	Mechanical Alignment	p-value
Pain score 0-10 (0=no pain / 10=worst pain)±SD	1.0±1.5	0.98±1.5	0.934
EQ-5d index 0-1 (0=worst health / 1=full health)±SD	0.90±0.1	0.91±0.1	0.535
Mean flexion in degrees (°)±SD	123±10.4	122±12.5	0.586

## Discussion

Our findings indicate the superiority of KA in TKR over MA for pain relief, knee flexion, and use of walking aids at six-weeks and three-months post-operation. These results are consistent with previous studies that highlighted the benefits of personalized alignment methods, with KA demonstrating improved outcomes regarding TKR, especially at early stages after surgery [7, 8, 10, 13].

While KA demonstrates clear benefits in the early postoperative period, our study suggests that the results of KA and MA converge over time, specifically at 12 months post-operation. This convergence highlights the dynamic nature of TKR outcomes and emphasizes the need for further research to explore the evolving impact of alignment techniques. The observed convergence contributes to the ongoing debate regarding the optimal alignment technique for TKR and the associated long-term implications. Further studies, involving larger cohorts and extended follow-up periods to fully determine the clinical impact of KA versus MA are warranted. Additionally, assessing various outcome measures, such as patient satisfaction, implant longevity, and functional performance, may provide a more comprehensive understanding of the impact of these alignment approaches.

While advancements in TKR technology have reduced joint replacement costs in recent years [15], comparative cost analyses among different surgical techniques remain important and necessary. Understanding the cost-effectiveness of various alignment methods, particularly when comparing KA with MA, is essential for tailoring the best approach to individual patients. Cost assessment including both perioperative and long-term expenses, is vital for informed decision-making in TKR surgery. 

The longevity and prospects of KA, its impact on healthcare costs, and the limitations of TKR are central to understanding its role in orthopedic surgery. Current data indicate comparable implant survival rates and clinical outcomes between total arthroplasty with kinematic alignment (KA-TKA) and total arthroplasty with MA-TKA. A remarkable 10-year survival rate of 97.5% was reported for more than 222 kinematically aligned knees, emphasizing the longevity of this procedure. However, compared to the long-established MA-TKA, which has an 80% 25-year survival rate, future long-term studies to fully validate the efficacy and durability of KA-TKA is justified [16,17].

Nevertheless, KA is not without limitations. It may be unsuitable for patients with extra-articular deformities or collateral ligament instability. Valgus deformities in particular pose a higher risk, so a moderate KA approach is recommended for optimal component positioning. Advanced preoperative planning, including weight-bearing radiographs of the long leg and three-dimensional anatomical assessments, is essential for assessing the suitability of KA and predicting postoperative outcomes [18].

The primary strength of this study lies in the consistency of the surgical approach: all surgeries were performed by a single experienced surgeon at the same institution using standardized implants. This consistency minimizes variability and enhances the reliability of the findings. Another strength is the exclusion of the transition year 2020, which reduces potential bias.

However, the study also has several limitations. Its retrospective design and single-center setting may limit the generalizability of the results. The relatively small sample size and lack of randomization could introduce selection bias. Importantly, our study focused on short-term outcomes, with follow-up limited to 12 months. To truly understand the long-term benefits and potential drawbacks of KA versus MA in TKR, larger, multicenter studies with randomized controlled designs and extended follow-up periods are essential. These studies should aim to evaluate not only clinical outcomes but also factors such as implant longevity, patient satisfaction over time, and long-term functional performance. By addressing these aspects, future research can provide a more comprehensive understanding of the long-term impact of alignment techniques in TKR.

## Conclusions

In this retrospective analysis comparing KA and MA in TKR, our results show significant short-term benefits associated with the KA, including enhanced pain relief, improved knee flexion, and reduced reliance on walking aids at six-weeks and three-months post-surgery. However, these initial advantages did not persist at the 12-month evaluation. Furthermore, there were no differences in patient-reported outcomes as measured by the EQ-5d questionnaire. The initial benefits of KA suggest its potential to provide improved functional outcomes and enhance rehabilitation during the early postoperative phase. Nevertheless, the observed convergence in outcomes between KA and MA at the 12-month follow-up underscores the complexity of selecting the ideal alignment technique for TKR, especially in the context of long-term patient satisfaction.

This study emphasizes the need for a balanced assessment of both short-term benefits and long-term outcomes when choosing the best alignment procedures for TKR. While KA may present certain postoperative advantages, the diminishing clinical significance of these differences over time highlights the need for an elaborate approach to surgical planning. Clinicians and patients are therefore encouraged to consider a variety of factors, including individual patient characteristics and expectations for long-term outcomes, when making informed decisions regarding the choice of alignment procedures for TKR. This comprehensive approach is essential for optimizing patient care and improving the overall success of TKR procedures.
